# Food Cue Reactivity Meets the Reinforcer Pathology Model: Behavioral Economic Measures of Cue-Induced Changes in Food Reinforcer Efficacy

**DOI:** 10.1007/s40614-024-00409-1

**Published:** 2024-07-02

**Authors:** Morgan Musquez, Erin B. Rasmussen

**Affiliations:** Psychology Department, Idaho State University, 921 South 8th Avenue, Pocatello, ID 83209 USA

**Keywords:** Binge eating, Delay discounting, Demand elasticity, Food cue reactivity, Obesity, Reinforcer pathology

## Abstract

Food cue reactivity, or behavioral sensitivity to conditioned food cues, is an eating pattern observed in those with obesity and binge-eating disorder. The reinforcer pathology model, which characterizes overconsumption of a reinforcer such as food may be relevant to food cue reactivity, especially in those with obesity and binge-eating disorder. The reinforcer pathology model posits that steep delay discounting (DD) and demand elasticity are processes involved in the overconsumption of food. Two of our recent studies examine the extent to which reactivity to conditioned food cues may be involved in food reinforcer pathologies. First, food cues were conditioned with Oreo cookies with binge-eating prone (BEP) and binge-eating resistant (BER) rats. Delay discounting was compared before and after conditioning. Food cues induced steeper DD for rats, though BEP rats showed some evidence for greater sensitivity to this effect than BER rats, albeit this difference was not significant. Second, healthy-weight humans and humans with overweight/obese BMI underwent conditioning of visual cues paired with M&M candies. After acquisition, cues induced greater demand intensity and inelasticity for food compared to baseline. Participants with overweight/obese BMI, compared to controls, also showed some evidence for greater sensitivity to this change ininelasticity compared to healthy-weight participants, but this difference was also not significant. Food cues, then, may induce changes in DD and economic demand, supporting the relevance of reinforcer pathologies.

Food cues are images of food, food-related odors, and food-related advertisements that are present in the environment. Food cues initially develop through the process of respondent (i.e., Pavlovian) conditioning—a type of learning in which a stimulus is paired with an unconditioned stimulus that elicits an unconditioned response (Jansen, 1998; Martin-Soelch et al., 2007; van den Akker et al., 2018). After a number of pairings, the paired stimulus begins to elicit a response as a conditioned stimulus (CS). Indeed, since the observations of Pavlov (1927), it has been widely known that environmental (e.g., sight or smell) or interoceptive cues (e.g., stress, negative affect, hormonal fluctuation, and food-related cognitions) that reliably signal food-related unconditioned appetitive responses, such as salivation or cephalic change (e.g., glucose or insulin production), may function as conditioned stimuli (Brede et al., 2017; Jansen, 1998; Jansen et al., 2003; Meyer et al., 2015; Nederkoorn et al., 2000; Rodin, 1985; Schüssler et al., 2012).

The presence of food cues also increases food consumption (Colagiuri & Lovibond, 2015; Versace et al, 2019) and self-reports of subjective food cravings and desire to eat (Nederkoorn et al., 2000; Tetley et al., 2009; van den Akker et al., 2013). The tendency for food cues to increase food consumption is well-supported by animal research (Petrovich et al., 2007; Reppucci & Petrovich, 2012; Weingarten, 1983). For example, Boggiano et al. (2009) respondently conditioned responses in rats by pairing a distinct “cookie cage” (CS) with Oreo Double Stuf cookies. Once food cue conditioning was complete, rats were placed in the “cookie” cage with access to only standard chow. Rats in the “cookie cage” (i.e., exposed to the food cue) consumed significantly more standard chow compared to a cage that was paired only with standard chow. The heightened food intake in the presence of the CS indicates greater conditioned appetitive responses, especially those paired with hedonic reward processes related to cookies.

There are individual differences regarding sensitivity to food cues, however—a phenomenon in the ingestion literature called *food cue reactivity* (Jansen, 1998). Food cue reactivity refers to physiological (e.g., saliva production, increased insulin levels and gastric activity), behavioral (e.g., food seeking and eating), and subjective (e.g., verbalizations of craving, urge, or desire to eat) responses to food-related stimuli (Jansen, 1998). The physiological responses prepare the organism for digestion, absorption, and metabolism of consumed nutrients and combined, all responses are involved in food seeking and consumption.

Food cue reactivity is positively related to food consumption (Jansen, 1998). Indeed, this general finding is summarized by a meta-analysis by Boswell and Kober (2016), which also links high food cue reactivity with obesity. A number of studies show that individuals with obese or overweight body status show higher food cue reactivity compared to controls (Hume et al., 2015; Jansen et al, 2003; Meyer et al., 2015; Tetley et al., 2009). Children and adults with obesity have higher cue-induced salivary flow (Jansen et al., 2003; Meyer et al., 2015), food consumption (Jansen et al., 2003), and self-reported desired portion sizes (Tetley et al., 2009).

Like individuals with obesity, those with binge-eating disorder (BED) are more sensitive to food cues than those without BED (Meule et al., 2018; Ng & Davis, 2013; Schienle et al., 2009; Svaldi et al., 2010). Binge-eating disorder is characterized by repetitive episodes (at least once a week for 3 months) of consuming large quantities of food in the absence of compensatory behaviors (e.g., vomiting, use of laxatives, or excessive exercise), eating until uncomfortably full, self-reported feelings of lack of control when eating, or reporting feelings of guilt or depression when eating (Agüera et al., 2021; de Zwaan, 2001). BED is not limited to those with obesity; however, it is the most common eating disorder reported among this population (Hudson et al., 2007; Kessler et al., 2013). It is also important to note that not everyone with obesity has BED. Nonetheless, in the presence of food cues, individuals with BED (compared to non-binge-eating controls) show increased food consumption (Ng & Davis, 2013) self-reported food cravings (Meule et al., 2018), greater neural processing of food stimuli (Svaldi et al., 2010), and enhanced brain activation in regions that reflect the hedonic value of food (Schienle et al., 2009).

Animal models of binge eating have been used to experimentally identify factors related to BED (see Avena, 2013; Turton et al., 2017). Binge-eating prone (BEP) rats consume larger amounts of highly palatable food (i.e., food high in sugar and fat content) in short periods of time (e.g., 1–4 hr) compared to binge-eating resistant (BER) rats (Boggiano et al., 2007, 2009); however, both groups consume similar amounts of less palatable food such as standard chow, indicating that highly palatable food may trigger binge-eating episodes (Boggiano et al., 2007). Indeed, highly palatable food has been shown to activate brain regions associated with hedonic food reward more in BEP rats than BER rats (Sinclair et al., 2015) and BEP rats will better tolerate foot shocks to gain access to highly palatable food compared to BER rats (Oswald et al., 2011).

## The Reinforcer Pathology Model

A reinforcer pathology refers to excessive valuation of an addictive (i.e., reinforcing) stimulus, such as a substance or food (Bickel et al, 2011; DeHart et al., 2020). Reinforcer pathologies involve two behavioral processes: (1) a persistently high valuation of a preferred commodity despite high response cost (i.e., inelastic demand); and (2) a consistently high preference for immediate consumption over delayed consumption of the commodity (i.e., delay discounting; Bickel et al., 2014). Demand elasticity refers to changes in consumption as a function of price (typically money or effort). In general, as price increases, the consumption of a reinforcer decreases. The less sensitive behavior is to price increases (called demand inelasticity), the more value the reinforcer has (Bickel et al., 2000; Hursh, 1980, 1984, 2000; Hursh & Silberberg, 2008; Madden, 2000).

Delay discounting (DD), the second behavioral process, refers to a decrease in the value of a reinforcer, such as food, drug, or money, as delay to its receipt increases (Odum, 2011). Delay discounting patterns are determined by presenting organisms with choices between smaller, immediate reinforcers versus larger, delayed reinforcers. A pattern of preference for smaller, more immediate outcomes indicates high sensitivity to delay (i.e., high or steep DD; Madden, 2000; Madden & Johnson, 2010; Odum, 2011, Odum et al., 2020).

Reinforcer pathologies have been characterized in individuals with substance use disorders (see Bickel et al., 2011, 2020; Jarmolowicz et al., 2016; McIntyre-Wood et al., 2022), but have also been applied to those with obesity (DeHart et al., 2020). Research with humans shows that individuals with obesity tend to overvalue food compared to lean individuals (Epstein et al., 2010b; Carr et al., 2011; DeHart et al., 2020). Regarding demand elasticity, those with obesity show less sensitivity to increases in effort for food compared to lean controls (Epstein et al., 2007; Giesen et al., 2010; Jacobs & Wagner, 1984; Saelens & Epstein, 1996; Temple et al., 2008). Obese nonhuman animals demonstrate greater demand inelasticity across increasing response requirements for food compared to leaner controls (Batten et al., 2020; Saelens & Epstein, 1996; Rasmussen et al., 2010b). Diet-induced obesity, in which obese-prone rats are fed high-fat diets, also predicts greater demand intensity (i.e., consumption at the lowest response cost) and inelasticity for food across increasing response requirements than obesity-resistant rats and rats fed low-fat diets (Batten et al., 2020).

Individuals with obesity also tend to have steep discounting. Human DD research with hypothetical food-related outcomes shows that individuals with obesity exhibit preferences for smaller, more immediate monetary and food rewards over larger, delayed ones when compared to leaner individuals (Amlung et al., 2016; Jarmolowicz et al., 2014; Lawyer et al., 2015; Rasmussen et al., 2010a; Schiff et al., 2016). Rodent models of obesity also indicate that obese rats discount delayed food more than lean controls (Boomhower et al., 2013; Robertson & Rasmussen, 2017).

Some studies show that DD is also a behavioral process involved in binge-eating behavior (Steward et al., 2017). When examining DD in humans, those with BED show steeper food (Manwaring et al., 2011) and monetary (Bartholdy et al., 2017) discounting than participants without BED. This effect is also observed with rats with binge-eating tendencies. Male Wistar rats that display higher food discounting rates are more prone to binge eating (Cano et al., 2016), and food discounting rates in female BEP Wistar rats are steeper than BER rats (Vickers et al., 2017).

The framework of the reinforcer pathology model could aid in understanding choice patterns in food cue reactivity, especially with regard to eating patterns that result in health risks. Food consumption is choice behavior (including what, when, and how much to consume) among a variety of other food- and non-food-related alternative reinforcers. A body of literature demonstrates that food consumption is heavily influenced by food availability in the environment. For instance, manipulating aspects of food availability such as the effort or price to obtain food and the delay to its receipt influences food intake. However, little to no research has examined the extent to which food cues may alter these aspects of food reinforcer processes. The present studies begin an examination of the extent to which conditioned food cues alter food cue reactivity by way of DD for food and demand elasticity for food in organisms with binge-eating behavior and obesity, respectively.

## Study 1: Conditioned Food Cues on Delay Discounting in a Rat Model of Binge Eating Subjects

Male Sprague-Dawley rats (*N* = 26) were purchased from Envigo (Livermore, CA, USA) at 3 weeks of age. Rats were housed in standard shoe-box style home cages and maintained on a 12:12 hr dark/light cycle, with lights on at 0700 hr. The Institutional Animal Care and Use Committee at Idaho State University approved all study procedures.

### Assignment of Binge-Eating Status

Binge-eating status was determined by procedures described in Boggiano et al. (2007, 2009), which capitalizes on the variation in binge eating observed in rats; this variation, incidentally, models the distribution of binge eating in humans. Briefly, rats were exposed to four 24-hr feeding sessions with access to highly palatable food (whole Oreo Double Stuf cookies) and standard chow. The average consumption of cookie and standard chow in kcals at the 4-hr time point was calculated across the four feeding sessions. The number of kcals was determined by multiplying the number of g consumed for both cookie and standard chow (g*4.8 kcal and g*3.6 kcal, respectively). Rats with the highest average cookie consumption were classified as binge-eating prone (BEP; *n* = 10); rats with the lowest average cookie consumption were classified as binge-eating resistant (BER; *n* = 10); the middle six rats were removed from the study. Average chow consumption also was measured to compare between BEP and BER rats during binge-eating assessment analysis, as foods with lower palatability tend to not differ significantly in BER and BEP rats; this measurement helps validate the binge model.

### Food Delay Discounting

Before food delay discounting (DD) could be assessed, rats were trained to lever press using methods similar to Boomhower et al. (2013). Food DD was measured using a two-lever procedure (Evenden & Ryan; 1996; Robertson & Rasmussen, 2017). One lever (left for half of the rats; right for the other half)—the smaller, sooner (SS) lever—resulted in an immediate delivery of one 45-mg pellet after a 0-s fixed delay. The opposite lever—the larger, delayed (larger, later; LL) lever—resulted in the delivery of three 45-mg pellets after a delay that increased systematically.

The procedures for DD sessions are described elsewhere (Evenden & Ryan, 1996; Robertson & Rasmussen, 2017) but are summarized here. A session was comprised of five trial blocks. Each trial block consisted of 2 forced-choice and 10 free-choice trials. In the forced-choice trials, the contingencies of the SS and LL lever were presented as two trials. First, one lever was presented, and the other was recessed. For example, the SS lever and associated cue light were presented. After the rat pressed the lever, the pellet was immediately delivered (0-s delay) and the cue light extinguished. An intertrial interval (ITI) of 60 s followed in which the lever retracted. Then the second trial began in which the other lever (the LL) and cue light were presented (the SS lever was recessed). When this lever was pressed, the cue light extinguished, three pellets were delivered after a specified delay, and an ITI followed in which the lever retracted. The ITI for each LL trial was dependent on the delay, such that the trial was 60 s (i.e., ITI was 60 s minus the delay). The presentation of SS and LL forced-choice trials was counterbalanced for order and placement (left or right) across rats.

**Fig. 1:**
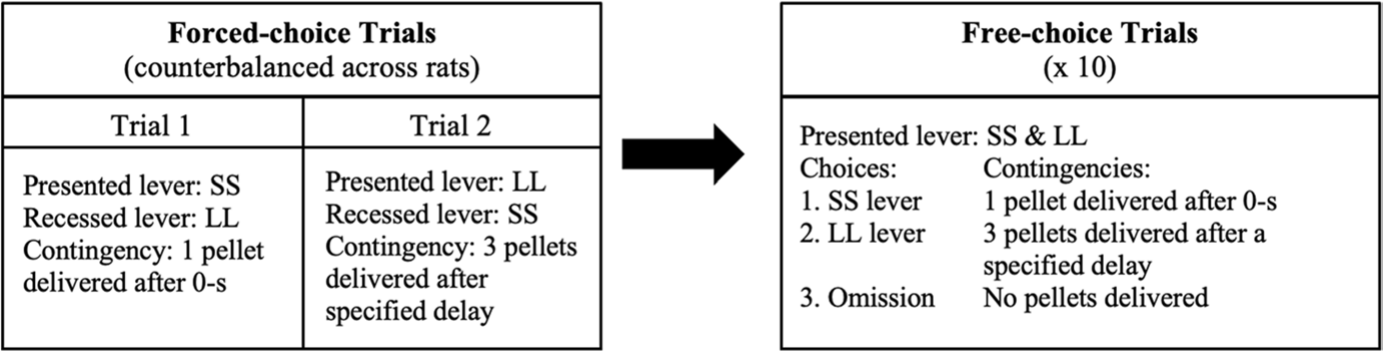
Trial Block Flow Chart for Food-Delay Discounting. *Note.* Each trial block consisted of 2 forced-choice and 10 free-choice trials for a specified delay. Because delay sequences contain five delays, five trial blocks (one for every delay) were executed per experimental session

Immediately after the forced-choice trials, there were 10 free-choice trials (see Fig. 1), each 60 s long. Here, both SS and LL levers were concurrently available with both stimulus lights on, in which rats could select: (1) the SS option; (2) the LL option; or (3) make no response after 30 s (an omission). If the SS lever was selected, one pellet was delivered after a 0-s delay and the cue light extinguished followed by a 60-s ITI in which the lever retracted. If the LL lever was selected, the cue light extinguished, three pellets were delivered after a specified delay, and then the ITI followed in which the lever retracted. If an omission occurred, both cue lights extinguished and both levers retracted for the remaining 30 s. There were 10 trials for each of the five delays, for a total of 50 trials across 5 trial blocks per each DD session. The first sequence of five delays to the LL was as follows: 0, 1, 2, 4, and 8 s. Delay discounting sessions were programmed for 1.5 hr.

### No-Cue (Baseline) Delay Discounting for Food

For the first delay sequence (0, 1, 2, 4, 8 s), if preference for the LL lever was > 50% on free-choice trials by the end of the session, the next higher delay sequence(s) was implemented in subsequent sessions (Sequence 2: 1, 2, 4, 8, 16 s; Sequence 3: 2, 4, 8, 16, 32 s; Sequence 4: 4, 8, 16, 32, 64 s; and Sequence 5: 8, 16, 32, 64, 128 s) until < 50% preference (preference reversal) was demonstrated. Each delay sequence was implemented for at least three sessions and continued until stability in behavior occurred. Stability was achieved when: (1) responses for the LL reinforcer did not show an increase or decrease across three consecutive sessions; and (2) responses for the LL reinforcer for a given session did not vary by more than 20% of the grand mean of the previous three sessions (see Robertson & Rasmussen, 2017). When delay sequences exceeded 60 s (Sequences 3 and 4), the ITI was the value of the last delay in the delay sequence. For example, if delay sequence #4 was implemented, the ITI was programmed for 64 s.

### Food Cue Conditioning and Cue-Exposed Chow Consumption

After no-cue DD was established, the food cue conditioning phase of the experiment commenced. For seven 24-hr sessions, each rat was placed in a “cookie” cage (i.e., separate from their home cage) that had been affixed with one of two stimuli: black construction paper along the sides of the cage or white construction paper placed over the bedding (see Boggiano et al., 2009, for more detail). Cookie cues were counterbalanced across groups. When in the “cookie” cage, highly palatable food (Oreo cookie), standard chow, and water were available ad libitum.

After the seven food-cue pairing sessions were complete, a test for conditioning to the cookie-cue commenced. Standard chow consumption was measured (in g) for two 24-hr periods in a cookie-cued versus no-cue (home cage) session. These two sessions occurred in counterbalanced order with 4 days between the sessions. In both 24-hr test sessions, rats received ad libitum standard chow and water. Chow consumption was measured at 4- and 24-hr time points.

### Cued Delay Discounting for Food

After the cues were conditioned, rats completed a second DD procedure in the same manner as the no-cue DD condition, except with the appropriate “cookie” cue present; that is, each operant chamber was affixed with the “cookie” cue that was conditioned during food cue training. Black construction paper was placed along the walls of the chamber or white construction paper was placed over the grid floor of the chamber, consistent with the cue to which each rat was assigned during the cue conditioning phase. To ensure the cookie cue did not lose its conditioned association to the cookie (i.e., extinction), rats were placed in the “cookie” cage with ad libitum Oreo cookie, standard chow, and water for single 24-hr sessions once a week until the cued DD phase was complete.

### Analyses

#### Delay Discounting for Food

Due to unstable responding during the no-cue DD session (decreasing trend across DD sessions), one BEP rat was excluded from DD analyses. Therefore, the final data set contained 19 subjects (BEP = 9, BER = 10). Food DD data were determined by plotting each rat’s percent of LL responses (mean of last three stable sessions) from the terminal delay sequence against delay (Robertson & Rasmussen, 2017). Linear best-fit functions were then fitted to each rat’s data and an indifference point for each rat was calculated by the delay value at which the best-fit function line corresponded to 50% LL response. The shorter the delay at indifference, the steeper the discounting rate.

#### Statistical Analyses

Data were analyzed using IBM SPSS 28.0©. Analyses for the 4-hr and 24-hr cookie-cued chow consumption consisted of separate 2 × 2 mixed ANOVAs, with binge-eating status (BEP vs. BER) as a between-subjects factor and cue condition (no-cue vs. cookie-cued) as a within-subjects factors. Analysis for DD consisted of a 2 × 2 mixed ANOVA, in which binge-eating status and cue condition were compared. Because there were no systematic differences between cookie cue type (black or white), cue data were pooled.

### Results

#### Binge-Eating Assessment

**Fig. 2:**
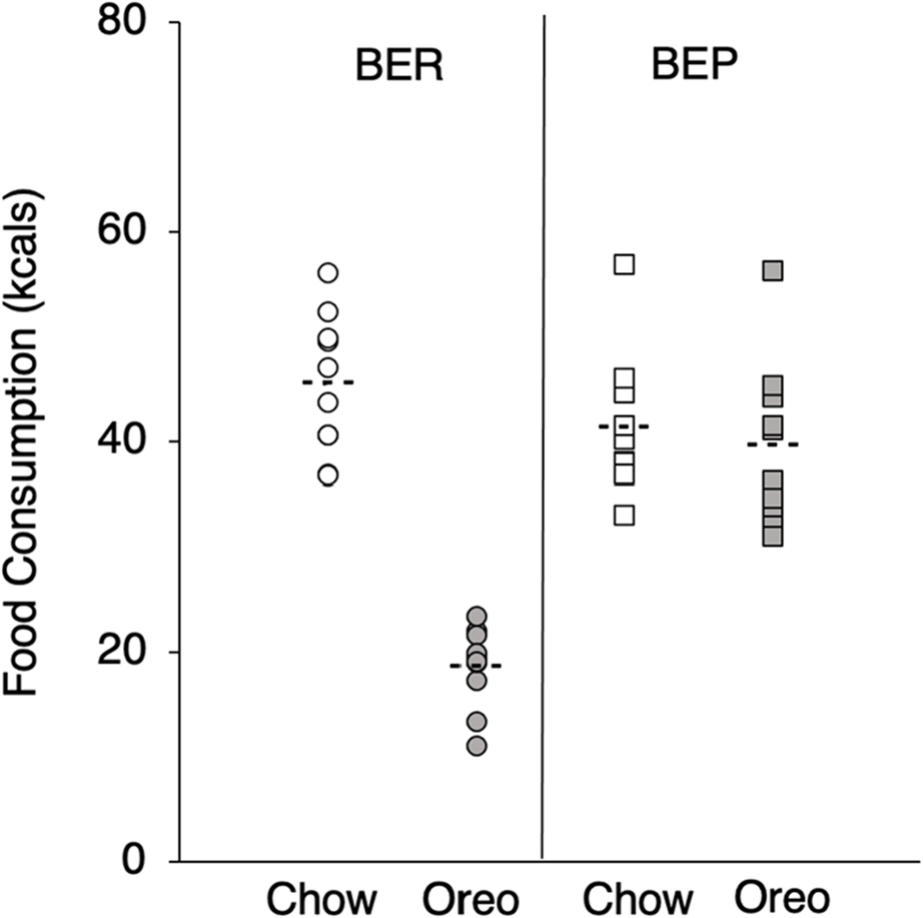
Individual Subject Data (Means = Dashed Lines) for Standard Chow (White) and Oreo Cookie (Grey) Intake in Kcals during the Binge-Eating Assessments as a Function of Binge-Eating Status

To show the variation in BEP (*n* = 10) and BER (*n* = 10) rats selected after the binge-eating assessment, Figure 2 shows standard chow and Oreo cookie consumption (in kcals) at the 4-hr time point. Binge-eating prone rats consumed an average of 39.64 kcals (*SEM* = 2.44) and 41.26 kcals (*SEM* = 2.11) from cookie and chow, respectively; binge-eating resistant rats consumed an average of 18.60 kcals (*SEM* = 1.22) and 45.34 kcals (*SEM* = 2.10) from cookie and chow, respectively. There were no significant differences for chow consumption between groups (*p* = .19). Due to the nature of the binge-eating assessment occurring in a small window of time, no significant differences in body mass (*p* = .31) were observed between BEP (*M* = 485.45 g, *SEM* = 16.96) and BER (*M* = 461.83 g, *SEM* = 14.80) rats.

#### Food Cue versus No-Cue on Chow Consumption at 4-hr and 24-hr time point.

**Fig. 3:**
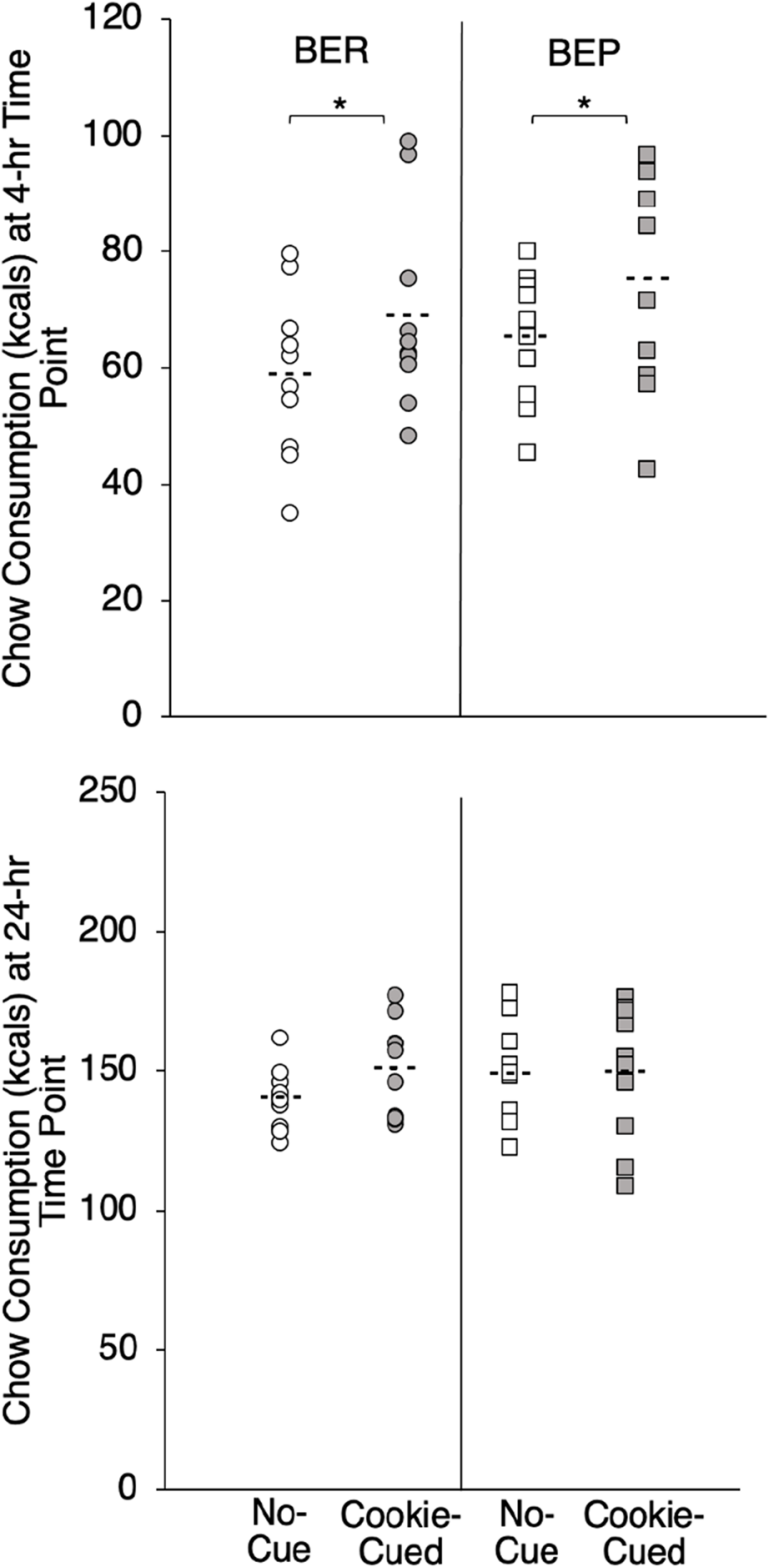
Individual Subject Data (Means = Dashed Lines) for Standard Chow Consumption as a Function of No-Cue (Baseline; White) versus Cookie-Cued (Grey) and Binge-Eating Status. *Note.* Top panel shows chow consumption at the 4-hr time point; bottom panel shows chow consumption at the 24-hr time point. * *p* < 0.05

Figure 3 shows chow intake (in kcals) at the 4-hr (top panel) and 24-hr (bottom panel) time points as a function of cue condition and binge-eating status. A 2 × 2 mixed ANOVA confirmed a main effect of cue condition on standard chow kcals consumed after 4 hrs (*F*(1, 18) = 12.91, *p* < .01, η^2^ = .42) but not after 24 hrs (*p* = .20). There were no main effects or interactions of binge-eating status for either time point (*p*’s > .29).

#### Food Cue Effects for Food Delay Discounting

**Fig. 4:**
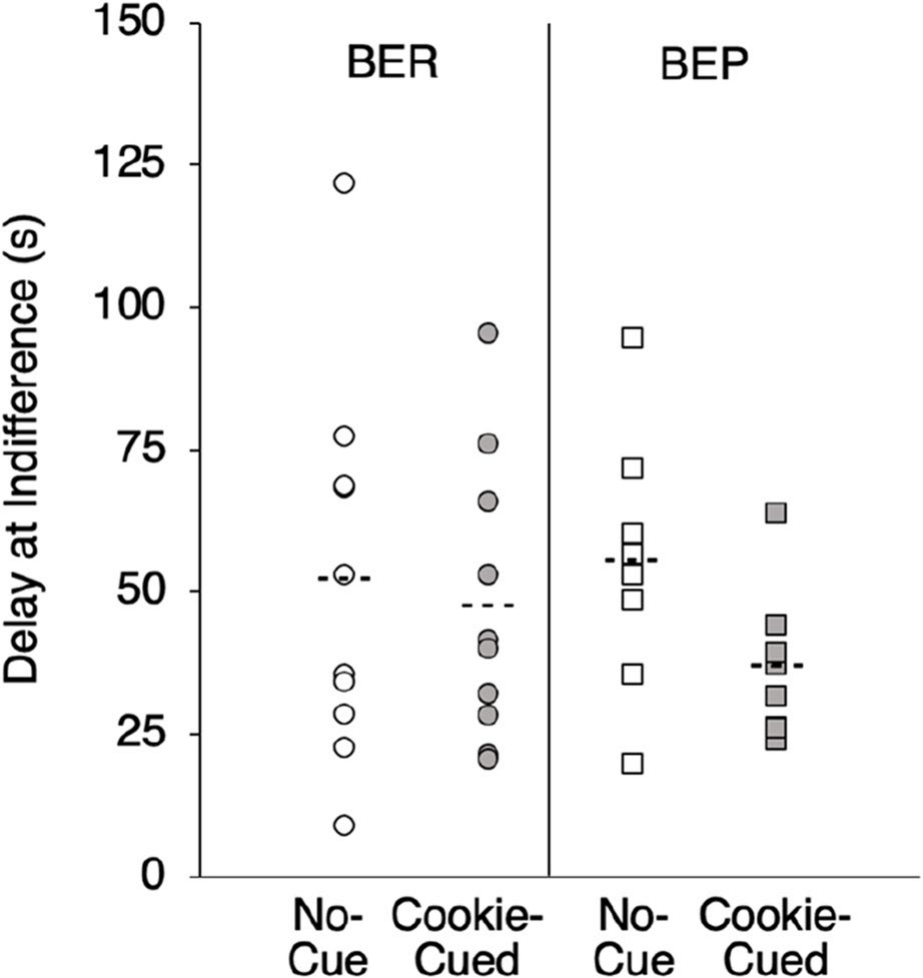
Individual Subject Data (Means = Dashed Lines) for Delay at Indifference (50% Preference for LL) as a Function of No-Cue (Baseline; White) versus Cookie-Cued (Grey) and Binge-Eating Status. *Note.* * *p* < 0.05

Figure 4 shows food DD values as measured by delay (s) at indifference as a function of cue condition and binge-eating status. A 2 × 2 mixed ANOVA revealed a main effect of cue condition on food DD, *F*(1, 17) = 6.17, *p* = .02, η^2^ = .27, with the cookie-cue inducing steeper discounting (i.e., lower delays at indifference) than the no-cue condition. There was no significant difference between groups (*p* = .72) nor an interaction (*p* = .15).

The R^2^ values for linear best-fit functions that determined delay (s) at indifference were satisfactory across conditions (mean R^2^ > .82). There were no significant differences of fit across group or cue condition (*p*’s > .30). No omissions occurred during any of the stable sessions in the no-cue or cookie-cued conditions.

### Discussion

Binge-eating prone rats consumed greater amounts of Oreo cookie than BER rats, but there were no significant group differences in body mass or standard chow intake reported by chow consumption during the binge-eating assessments and home cage chow consumption test, replicating previous literature using this binge-eating model (Boggiano et al., 2007, 2009). Therefore, this animal model of binge-eating behavior appeared valid and consistent with other studies.

Conditioned food cues increased chow consumption at the 4-hr time point for BEP and BER rats compared to noncued conditions. These results add to the body of literature in which conditioned food cues increase food consumption in rats (Boggiano et al., 2009; Reppucci & Petrovich, 2012; Weingarten, 1983). This effect, however, was not observed at the 24-hr time point. Replicating Boggiano et al. (2009), we found no significant group differences in cookie-cued standard chow consumption at either the 4- or 24-hr time points; BEP and BER rats consumed similar amounts of chow across the food cue test sessions. This finding may initially seem counterintuitive, given literature that suggests organisms with binge-eating behaviors are more reactive to food cues than those without binge-eating behaviors (Meule et al., 2018; Ng & Davis, 2013; Schienle et al., 2009; Svaldi et al., 2010). However, those with binge-eating behaviors typically overconsume *highly palatable food* and not less palatable foods. Studies with humans examining nutrient content during a binge-eating episode have shown that those who binge eat consume a greater percentage of energy from fat (Bartholome et al., 2013; Raymond et al., 2012; Yanovski et al., 1992) and sugar (Dalton et al., 2013; Hadigan et al., 1989) compared to those who do not binge eat. Unfortunately, our study did not include a condition that tested for food cue-induced consumption of foods that differed in palatability. Future research should replicate the current study using foods with different nutrient content, especially those that differ in sugar or fat content to more fully characterize cue-related effects on foods of differing palatability.

Food-cue exposure also altered DD for food. Delays at indifference were statistically shorter in duration after cue exposure compared to baseline. This suggests that conditioned food cues induced greater DD for food compared to baseline. To our knowledge, this is the first report of food cue-induced changes in food DD in rats. It did appear the BEP rats showed a stronger sensitivity to cues, though this did not manifest in a significant group difference or interaction. Nonetheless, these results add to the food cue literature by providing additional support in which food cues alter food choices by shifting food preferences toward the smaller, more immediate option.

We did not observe significant differences in food DD rates between BEP and BER rats, which does not support previous literature (Cano et al., 2016; Vickers et al., 2017). One major difference between the current study and the others, however, was the use of grain-based pellets in the discounting task whereas other studies used sucrose pellets. This difference offers additional support for using foods that differ in palatability. Nonetheless, despite the use of standard grain pellets, our findings suggest that conditioned food cues induce steeper DD and higher chow consumption.

This study, then, supports that food cue reactivity may be related to the reinforcer pathology model in which conditioned food cues not only induce greater discounting. The extent to which conditioned food cues alter other aspects of the reinforcer pathology model was explored in a study with humans.

## Study 2: Effects of Conditioned Food Cues on Salivation and Elasticity of Demand in Humans with Overweightness and Obesity

### Participants

Female (*N* = 47) college students enrolled in lower-division psychology courses were recruited from Idaho State University via SONA—an online subject pool. Sample size was determined by an a priori power analysis with an effect size = .25; a sample of 44 participants (22 healthy-weight, 22 overweight/obese) resulted in α = .05 and power = .80. Exclusion criteria included a current or past diagnosis (within 2 years) of an eating disorder, current pregnancy, and/or a diagnosis of diabetes. Participants were asked not to eat or drink for at least 2 hr before each session. The Institutional Review Board at Idaho State University approved all study procedures.

### Most Relevant Measures and Materials

#### Demand

The Food Purchasing Task (FPT; *α* = .84; Epstein et al., 2010a) is a demand-based questionnaire-style measure of food reinforcer efficacy. Participants are asked to indicate the number of portions they would be willing to purchase for a preferred food item at 18 different prices ranging from $0.01 to $1,120. To standardize portions, participants were given a 5/8-in cube and asked to imagine it as one portion of their favorite food prior to administering the questionnaire.

#### Swallowing

Prior research has shown that swallowing and salivary response are highly correlated (Nederkoorn et al., 1999), therefore, swallowing was used as a proxy for salivation. Swallowing was measured using an electromyograph (EMG) and recorded at 250 Hz using a SR-Lab EMG amplifier (San Diego Instruments, San Diego, CA). Unfortunately, because of measurement error, the data from conditioned swallowing were uninterpretable and no analyses were able to be performed.

#### Obesity

To determine obesity status, participants’ height and waist circumference were measured and collected in centimeters (cm) using a standard measuring tape. Body Mass Index (BMI) was determined by the ratio of a participant’s body mass in kilograms to the square of their height measured in meters. Participants with a BMI < 25 were considered healthy-weight; participants with a BMI > 24.9 were classified as overweight/obese.

### Procedure

#### Session 1

Each participant arrived at the Health Decisions Laboratory at Idaho State University and was escorted to an office-sized room. After obtaining informed consent, researchers attached three electrodes to the participant (two under the jaw about 1 cm apart and one behind the left ear on the mastoid bone) to measure swallowing as a proxy for salivation.

To collect baseline swallowing data to neutral cues, participants were asked to sit in a stationary chair and watch a computer screen that displayed a neutral stimulus (colored shape, such as a yellow triangle) for 180 s. The researcher recorded the exact time of any activity that could disrupt accurate measurement of swallowing (e.g., coughing, sneezing, or verbalizations). Participants then completed the Food Purchasing Task (FPT) while the computer screen displayed the neutral stimulus to establish baseline demand intensity and elasticity for food.

Participants then completed the acquisition procedure in which they were presented with 20 conditioning (CS+) trials for the stimulus used during baseline and 20 inhibitory conditioning trials (CS-) for a second visual stimulus (e.g., blue triangle) via computer screen. The assignment of stimuli to each participant was randomized from six colored shapes (e.g., blue triangle, red square, yellow circle, orange rectangle, green oval, and purple pentagon).

**Fig. 5:**
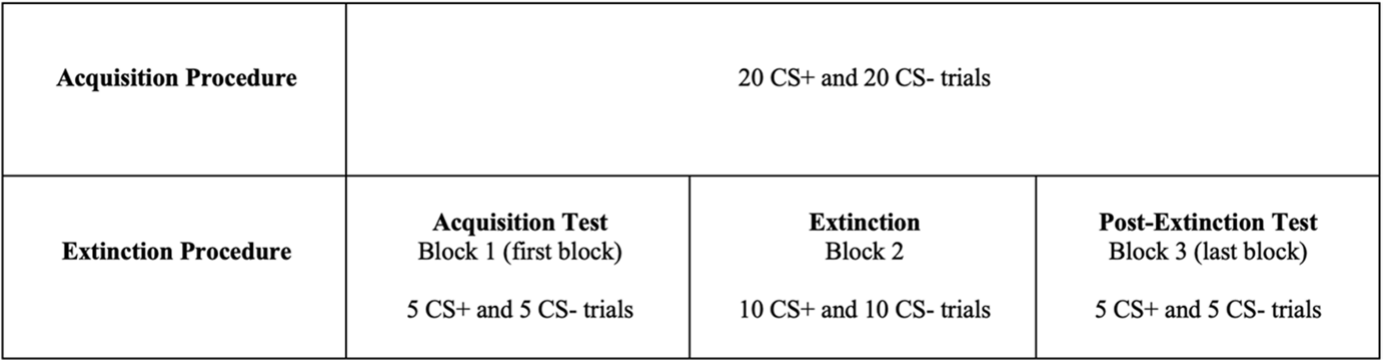
Visual Representation of the Acquisition and Extinction Paradigm. *Note.* During the acquisition procedure in Session 1, 20 CS+ and 20 CS- trials were administered. In session 2, during the extinction procedure (also 20 trials but with no US), the acquisition test consisted of presentation of the first 5 CS+ and 5 CS- trials (first block). This was followed by 10 CS+ and 10 CS- trials. The postextinction test consisted of the last 5 CS+ and 5 CS- trials (last block)

Figure 5 describes acquisition trials for CS+ and CS- stimuli, which were programmed in E-Prime based on procedures described in Meyer et al. (2015). During CS+ trials, the computer screen displayed a shape (the same shape the participant received during baseline) for 7.5 s, which was followed by an unconditioned stimulus (US) delivery. For US delivery, the computer screen instructed the participants to eat one M&M. Participants were given 10 s to consume the bite of food in which the computer screen displayed a 10 s countdown of the time remaining to consume the US. After 10 s elapsed, there was an 18.5-s intertrial interval (ITI) that followed in which the computer displayed a blank screen. Thus, the total time for a CS+ trial was 36 s. The CS- trials were the same, except following the presentation of a different shape (the CS-), no US was delivered. The CS- stimulus was presented for 7.5 s. To ensure consistency with CS+ trials, a 28.5 s ITI followed the CS- presentation, such that the total time for a CS- trial was 36 s. The presentation of CS+ and CS- trials were alternated throughout the procedure.

After completing the acquisition procedure, researchers collected information about the participants’ heights and weights. Body Mass Index was then calculated using the biometric measurements. A second session was scheduled for the following week.

#### Session 2

Participants arrived at the same location as Session 1 and completed the FPT in the presence of the CS+ to establish post-acquisition demand intensity and elasticity for food. Next, electrodes were placed on the participant in the same manner as Session 1 to assess acquisition of the conditioned stimulus and then to begin the extinction procedure.

#### Testing for Acquisition and Extinction Procedure

The test for acquisition of the CS and the implementation of extinction were conducted using the same acquisition procedures from session 1, except there were no M&Ms (no US) delivered during the CS+ trials. To determine conditioning of the CS+, swallowing rate was recorded during the first block (i.e., the first 10 trials) of the extinction procedure, in which 5 CS+ and 5 CS- trials were presented (see Fig. 5). The total number of swallows was counted for both CS+ and CS- in this first block.

Each extinction trial consisted of presenting a CS+ for 7.5 s with no food deliveries or instructions for eating. After the 7.5 s CS+ presentation, a 28.5 s ITI commenced, during which the computer displayed a blank screen. The same procedure was used for CS- trials, and 40 (20 for CS+ and 20 for CS-) extinction trials took place. Consistent with Meyer et al. (2015), CS- stimuli were extinguished as well to maintain consistency with previous trials and ensure the participant remained naïve to the purpose of the experiment. Like the acquisition procedure, the presentation of CS+ and CS- trials were alternated throughout the procedure.

To test for extinction, swallowing was recorded during the final block (i.e., the last 10 trials) of the extinction procedure. Like the acquisition test, each participant received 5 CS+ and 5 CS- trials during the final block. The total number of swallows was counted for both CS+ and CS-. After completion of the extinction trials, the participants were instructed to complete the FPT as post-extinction demand intensity and elasticity for food, during which the computer screen displayed the CS+ image.

### Analyses

#### Demand

To measure demand elasticity and intensity, the exponential model of demand (Hursh & Silberberg, 2008; Eq. 1) was fitted to the data from the FPT for each participant using nonlinear regression:

1 $$\text{log}Q={Q}_{0}+k\left({e}^{-\alpha {Q}_{0}^{P}}-1\right)$$

Here, *Q* refers to the number of reinforcers bought at a given price, *P*. *Q*_*0*_ is consumption at the lowest price (i.e., demand intensity), *k* is a constant representing the range of the dependent variables in logarithmic units, and *α* is the parameter that describes the slope of exponential decline in demand, i.e., elasticity or sensitivity to price. Reinforcers that have relatively high *α* values have higher elasticity (i.e., sensitive to price increases), whereas reinforcers with relatively low *α* values are considered to be inelastic (i.e., insensitive to price increases).

Free parameter values for *Q*_*0*_ and *α* were estimated for each participant. All demand data were inspected for relative fitness to the exponential model of demand equation, and if R^2^ values for a participant were < .70, participants were removed from demand analyses (Rasmussen et. al, 2010b). This resulted in two participants being excluded. Therefore, the final data set contained 45 participants (healthy-weight = 23, overweight/obese = 22). Due to the skewness of the distribution, *α* and *Q*_*0*_ values were log_10_ transformed.

#### Statistical Analyses

Data were analyzed using IBM SPSS 28.0©. Main analyses consisted of 2 × 3 mixed ANOVAs, with obesity status (healthy-weight vs. overweight/obese) as the between-subjects factor and conditioning phase (baseline, postacquisition, postextinction) as the within-subjects factor for CS+ data only. Mauchly’s Test of Sphericity was significant for demand intensity and demand elasticity; thus, the Greenhouse-Geisser correction was used.

## Results

### Participant Demographics

Most of the sample (*N* = 47) was white (77%) and college-aged (*M* = 21.49, *SEM* = .95). Of the total 47 women, 24 (51%) were classified as healthy-weight and 23 were classified with overweightness/obesity. As expected, several health-related differences were found between groups. Women with overweightness/obesity weighed more (*t*(45) = 8.24, *p* < .001, *d* = 2.40) and had higher BMIs (*t*(45) = 8.04, *p* < .001, *d* = 2.35), percent body fat (*t*(45) = 10.34, *p* < .001, *d* = 3.02), and waist circumferences (*t*(45) = 8.11, *p* < .001, *d* = 2.37) relative to healthy-weight woman. No other significant differences were observed.

### Demand

Mean R^2^ values for the exponential model of demand were satisfactory across conditions and group (R^2^ means > .91). There were no significant differences of fit across group or condition (*p*’s > .20).

#### Demand Intensity

**Fig. 6:**
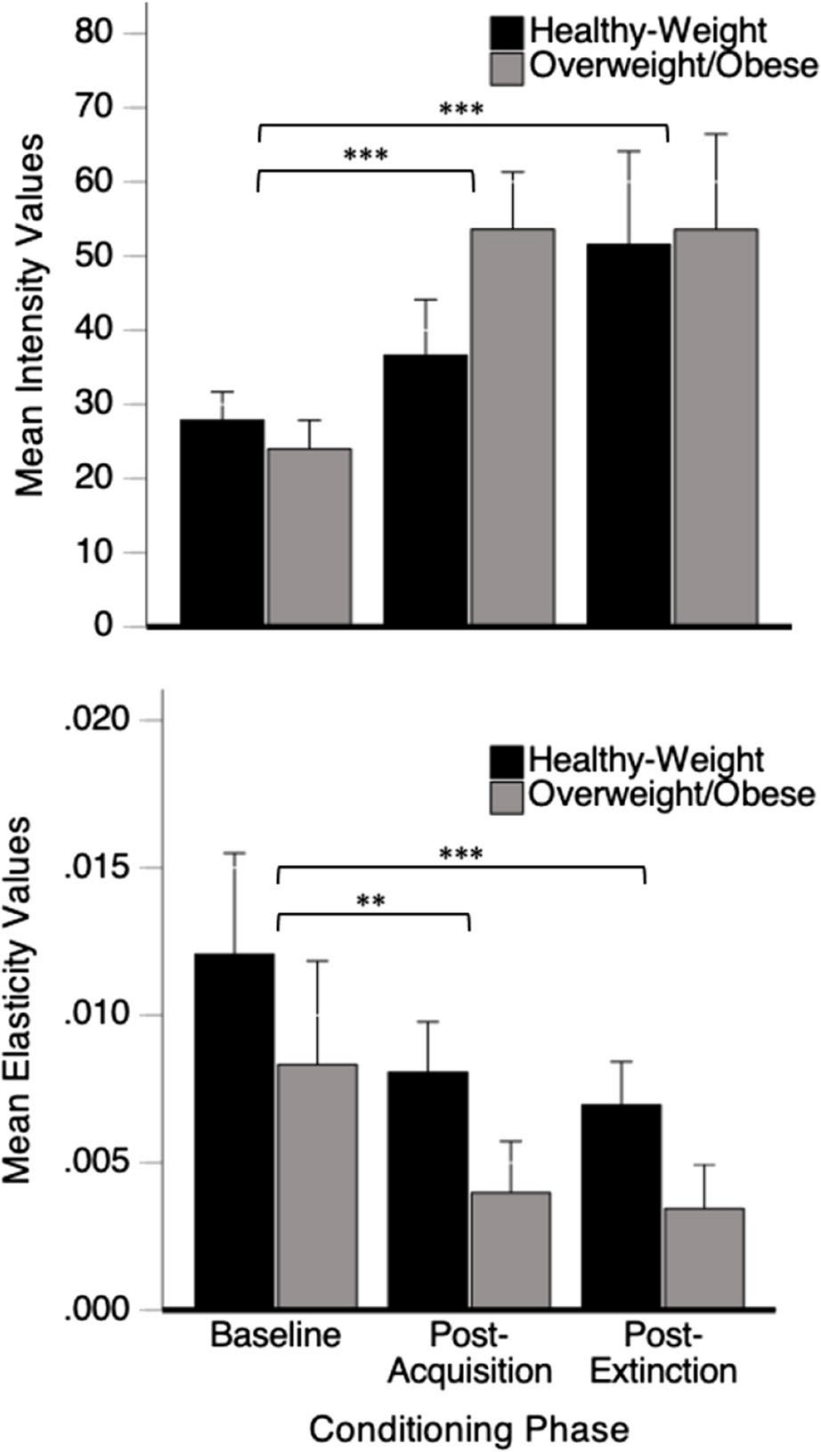
Mean Intensity (*Q*_*0*_ Values; Top) and Demand Elasticity (*α* Values; Bottom) as a Function of Condition and Obesity Status (Healthy-Weight = Black; Overweight/Obese = Grey). *Note.* Error bars represent 1 SEM.** *p* < 0.01; *** *p* < 0.001

The top of Fig. 6 shows mean demand intensity (*Q*_*0*_ values; log_10_-transformed) as a function of conditioning phase and obesity status. A 2 x 3 mixed ANOVA revealed a main effect of condition on *Q*_*0*_ values, *F*(1.53, 65.76) = 34. 64, *p* < .001, η^2^ = .45. Post-hoc contrasts revealed that intensity values were significantly higher post-acquisition and during extinction compared to baseline (*p*’s < .001). There was no main effect of obesity status (*p* = .47), and there was a marginal interaction between condition and obesity status (*p* = .09).

#### Demand Elasticity

The bottom of Fig. 6 shows mean demand elasticity (*α* values) as a function of conditioning phase and obesity status. A 2 x 3 mixed ANOVA revealed a main effect of condition (*F*(1.25, 53.76) = 11.52, *p* = .001, η^2^ = .21) and a marginal effect of obesity status on *α* values (*p* = .08) but no interaction (*p* = .37). All participants showed a decrease in elasticity across conditions, as post-hoc contrasts revealed that *α* values at post-acquisition and post-extinction were significantly lower than baseline (*p*’s < .01). Post-extinction elasticity values were also significantly lower than post-acquisition values (*p* < .01).

### Discussion

Because of measurement error, we were unable to get conclusive data on the conditioned swallowing (salivation) measure. Nonetheless, following acquisition, food cue exposure altered demand elasticity (sensitivity to effort) and intensity (consumption at the lowest price) for both healthy-weight participants and participants with overweightness/obesity. In particular, our results indicate that conditioned food cues decreased elasticity of demand—making demand for food more inelastic. In other words, higher prices for food were tolerated when food cues were presented. In addition, conditioned food cues increased demand intensity, inducing more consumption of food at lower prices. These data suggest that the conditioned food cues potentiated the reinforcing properties of food at both lower and higher prices. To our knowledge, this is the first report of food cue-induced changes in demand elasticity and intensity with food cues that are conditioned in a laboratory setting.

The changes in demand elasticity and intensity were not reduced after the extinction paradigm, providing support for the use of an extended extinction procedure (i.e., using more trials for extinction). It is interesting that, following extinction, participants demonstrated even greater inelasticity across increasing prices) compared to baseline and postacquisition. This finding was unexpected and suggests that the extinction procedure—food cue exposure without M&M reinforcement—further increased inelasticity for food. It is also possible that participants may be sensitive to deprivation effects. Because the extinction procedure took 24 min to complete, one hypothesis is that deprivation of food across extinction played a role in increasing demand inelasticity. Indeed, other studies with deprivation and elasticity have shown that demand is more inelastic following periods of deprivation or withdrawal (Jensen et al., 2004; and Wade-Galuska et al., 2011, respectively). One way to control for deprivation would be to test early in the experimental session after extinction has ensued. A simple way to do this might be to conduct the extinction trials in one session and then test the CS+ for extinction at the beginning of a new session the next day. Using multiple sessions could reduce retention of participants, however.

Extinction of food cue-related stimuli depends highly on the extent to which someone will refuse food when the cue is presented, which may be unlikely for some. Even if rejection (i.e., not eating) of food occurs, it is clear many trials would still be needed to extinguish a food cue. The number of trials, however, is not well-characterized. Because there were not enough extinction trials in the current study, researchers could design future studies that examine extinction of food cues to completion that would be individualized for each participant. Resistance to extinction of conditioned food cues may be an important aspect of obesity status as well.

There were no statistically significant main effects (though perhaps marginal effects) of overweight/BMI obesity status in the demand data or an interaction between group and cue conditioning. The data suggest that perhaps BMI might contribute to driving the cue conditioning effects, but is not a significant contributor per se. It may also be the case that combining overweight participants with those in the obese group may have “washed out” differences between those with healthy weight versus obese BMIs. More research on comparing groups with greater differences in BMI is recommended.

This study was a first attempt at examining the degree to which food cues might affect elements of demand elasticity. As such there were some places for improving the methods. First, testing for elasticity effects with the CS- would be important to determine whether the effects of the conditioned cues were specific to the CS+. Second, as mentioned, our study was also limited in terms of allowing enough trials for extinction to occur. Another limitation included the use of only female participants, which were used due to electrode placement and the absence of facial hair. Gender differences in food cue reactivity may be possible, but they are not supported by a meta-analysis (Boswell & Kober, 2016), in which mixed-gender samples yield similar results as female-only samples. Nonetheless, future research should replicate and extend this study by including a more diverse sample.

Overall, our results suggest that conditioned food cues potentiate food reinforcer efficacy as measured by demand intensity and elasticity. Though these data are limited by some methodological factors that can certainly be refined with additional studies, we believe they represent some promise with regard to examining the extent to which food cues can induce transient changes in elasticity of demand for food.

## General Discussion and Implications

These are the first studies to show that conditioned food cues change behavioral processes involved with reinforcer pathologies related to food. Results from the first study suggest that conditioned food cues induce immediate chow consumption and potentiate food delay discounting (DD), both indicators of urgency in eating. Binge-eating prone (BEP) rats may have shown some sensitivity to these cues, but more data are needed. There are implications to these findings. Conditioned food cues appear to induce behavioral processes for immediate food consumption (i.e., food urgency), which may generalize to contexts in which food cues are ubiquitous and may influence immediate food choices. Countries and contexts in which fast food is ubiquitously advertised (cues) and food is available more readily may lead to food-urgent behavior. This indeed has been shown in obesogenic environments (Boomhower et al., 2013; Lawyer et al., 2015; Rasmussen et al., 2010a; Schiff et al., 2016).

Results from the second study with humans suggest that conditioned food cues potentiate food reinforcer efficacy as measured by demand elasticity and intensity. This too is a novel and important finding, with obesity status potentially playing a role. There are several implications to these findings. Once, conditioned food cues may not only momentarily alter reinforcer value (as measured by economic demand), but this effect may be persistent and resistant to extinction. Our results suggest that extinction of food cues and the potential effects on demand elasticity and intensity may take substantially longer than the acquisition of food cues. These findings replicate and extend the food cue literature beyond food intake and cravings by identifying processes associated with food accessibility by way of price.

Overall, the data support that conditioned food cues affect processes related to reinforcer pathologies. However, there were some limitations. First, including both aspects of reinforcer pathologies—demand elasticity and DD—in both studies may have provided more meaningful implications. Second, variables such as diet history, especially those with high refined carbohydrate content (Avena et al., 2008), and dietary restraint (Hagan & Moss, 1997), may also be factors that affect the behavioral processes in this study. Indeed, animal research shows that high-fat/high-sugar diets blunt reward processes and increase food intake while altering dopaminergic signaling (Fritz et al., 2018; Robertson & Rasmussen, 2017; Shafat et al., 2009). Therefore, future research should examine the extent to which these factors may affect food-cue reactivity, DD, and demand elasticity for food. Finally, researchers may consider examining these processes with individuals with both BED and obesity; this particular sample may be especially likely to demonstrate shifts in food cue reactivity, DD, and demand elasticity. Resistance to extinction to food cues also needs further characterization in individuals with these characteristics.

## Data Availability

The datasets generated during and/or analyzed during the current study are available in this data repository: FCR and Reinforcer Pathology.
